# Association of objective sleep duration with cognition and brain aging biomarkers in older adults

**DOI:** 10.1093/braincomms/fcae144

**Published:** 2024-04-26

**Authors:** Shi Tang, Rui Liu, Juan Ren, Lin Song, Lingling Dong, Yu Qin, Mingqing Zhao, Yongxiang Wang, Yi Dong, Tong Zhao, Cuicui Liu, Tingting Hou, Lin Cong, Shireen Sindi, Bengt Winblad, Yifeng Du, Chengxuan Qiu

**Affiliations:** Department of Neurology, Shandong Provincial Hospital Affiliated to Shandong First Medical University, Jinan 250021, China; Department of Neurology, Shandong Provincial Hospital, Shandong University, Jinan 250021, China; Shandong Provincial Clinical Research Center for Neurological Diseases, Jinan 250021, China; Medical Science and Technology Innovation Center, Shandong First Medical University & Shandong Academy of Medical Sciences, Jinan 250117, China; Department of Ultrasound, Shandong Provincial Hospital Affiliated to Shandong First Medical University, Jinan 250021, China; Department of Neurology, Shandong Provincial Hospital, Shandong University, Jinan 250021, China; Shandong Provincial Clinical Research Center for Neurological Diseases, Jinan 250021, China; Department of Neurology, Shandong Provincial Hospital Affiliated to Shandong First Medical University, Jinan 250021, China; Department of Neurology, Shandong Provincial Hospital, Shandong University, Jinan 250021, China; Shandong Provincial Clinical Research Center for Neurological Diseases, Jinan 250021, China; Medical Science and Technology Innovation Center, Shandong First Medical University & Shandong Academy of Medical Sciences, Jinan 250117, China; Department of Neurology, Dongying People’s Hospital, Dongying 257091, China; Department of Neurology, Liaocheng People’s Hospital, Liaocheng 252000, China; Department of Neurology, Shandong Provincial Hospital Affiliated to Shandong First Medical University, Jinan 250021, China; Shandong Provincial Clinical Research Center for Neurological Diseases, Jinan 250021, China; Department of Neurology, Shandong Provincial Hospital Affiliated to Shandong First Medical University, Jinan 250021, China; Department of Neurology, Shandong Provincial Hospital, Shandong University, Jinan 250021, China; Shandong Provincial Clinical Research Center for Neurological Diseases, Jinan 250021, China; Medical Science and Technology Innovation Center, Shandong First Medical University & Shandong Academy of Medical Sciences, Jinan 250117, China; Institute of Brain Science and Brain-Inspired Research, Shandong First Medical University & Shandong Academy of Medical Sciences, Jinan 250117, China; Aging Research Center and Center for Alzheimer Research, Department of Neurobiology, Care Sciences and Society, Karolinska Institutet-Stockholm University, 171 65 Solna, Sweden; Department of Neurology, Shandong Provincial Hospital Affiliated to Shandong First Medical University, Jinan 250021, China; Department of Neurology, Shandong Provincial Hospital, Shandong University, Jinan 250021, China; Shandong Provincial Clinical Research Center for Neurological Diseases, Jinan 250021, China; Medical Science and Technology Innovation Center, Shandong First Medical University & Shandong Academy of Medical Sciences, Jinan 250117, China; Department of Neurology, Shandong Provincial Hospital, Shandong University, Jinan 250021, China; Shandong Provincial Clinical Research Center for Neurological Diseases, Jinan 250021, China; Department of Neurology, Shandong Provincial Hospital Affiliated to Shandong First Medical University, Jinan 250021, China; Department of Neurology, Shandong Provincial Hospital, Shandong University, Jinan 250021, China; Shandong Provincial Clinical Research Center for Neurological Diseases, Jinan 250021, China; Medical Science and Technology Innovation Center, Shandong First Medical University & Shandong Academy of Medical Sciences, Jinan 250117, China; Department of Neurology, Shandong Provincial Hospital Affiliated to Shandong First Medical University, Jinan 250021, China; Department of Neurology, Shandong Provincial Hospital, Shandong University, Jinan 250021, China; Shandong Provincial Clinical Research Center for Neurological Diseases, Jinan 250021, China; Medical Science and Technology Innovation Center, Shandong First Medical University & Shandong Academy of Medical Sciences, Jinan 250117, China; Department of Neurology, Shandong Provincial Hospital Affiliated to Shandong First Medical University, Jinan 250021, China; Department of Neurology, Shandong Provincial Hospital, Shandong University, Jinan 250021, China; Shandong Provincial Clinical Research Center for Neurological Diseases, Jinan 250021, China; Medical Science and Technology Innovation Center, Shandong First Medical University & Shandong Academy of Medical Sciences, Jinan 250117, China; Aging Research Center and Center for Alzheimer Research, Department of Neurobiology, Care Sciences and Society, Karolinska Institutet-Stockholm University, 171 65 Solna, Sweden; Division of Clinical Geriatrics, Department of Neurobiology, Care Sciences and Society, Karolinska Institutet, 171 64 Solna, Sweden; Neuroepidemiology and Ageing Research Unit (AGE), School of Public Health, Imperial College London, London SW7 2AZ, United Kingdom; Division of Neurogeriatrics and Center for Alzheimer Research, Department of Neurobiology, Care Sciences and Society, Karolinska Institutet, 171 64 Solna, Sweden; Theme Inflammation and Aging, Karolinska University Hospital, 141 83 Huddinge, Sweden; Department of Neurology, Shandong Provincial Hospital Affiliated to Shandong First Medical University, Jinan 250021, China; Department of Neurology, Shandong Provincial Hospital, Shandong University, Jinan 250021, China; Shandong Provincial Clinical Research Center for Neurological Diseases, Jinan 250021, China; Medical Science and Technology Innovation Center, Shandong First Medical University & Shandong Academy of Medical Sciences, Jinan 250117, China; Institute of Brain Science and Brain-Inspired Research, Shandong First Medical University & Shandong Academy of Medical Sciences, Jinan 250117, China; Department of Neurology, Shandong Provincial Hospital Affiliated to Shandong First Medical University, Jinan 250021, China; Institute of Brain Science and Brain-Inspired Research, Shandong First Medical University & Shandong Academy of Medical Sciences, Jinan 250117, China; Aging Research Center and Center for Alzheimer Research, Department of Neurobiology, Care Sciences and Society, Karolinska Institutet-Stockholm University, 171 65 Solna, Sweden

**Keywords:** sleep duration, mild cognitive impairment, neurodegeneration, brain aging, population-based study

## Abstract

The neuropathological mechanisms underlying the association between sleep duration and mild cognitive impairment remain poorly understood. This population-based study included 2032 dementia-free people (age ≥ 60 years; 55.1% women) derived from participants in the Multimodal Interventions to Delay Dementia and Disability in Rural China; of these, data were available in 841 participants for Alzheimer’s plasma biomarkers (e.g. amyloid-β, total tau and neurofilament light chain), 1044 for serum microvascular biomarkers (e.g. soluble adhesion molecules) and 834 for brain MRI biomarkers (e.g. whiter matter, grey matter, hippocampus, lacunes, enlarged perivascular spaces and white matter hyperintensity WMH). We used electrocardiogram-based cardiopulmonary coupling analysis to measure sleep duration, a neuropsychological test battery to assess cognitive function and the Petersen’s criteria to define mild cognitive impairment. Data were analysed with multivariable logistic and general linear models. In the total sample (*n* = 2032), 510 participants were defined with mild cognitive impairment, including 438 with amnestic mild cognitive impairment and 72 with non-amnestic mild cognitive impairment. Long sleep duration (>8 versus 6–8 h) was significantly associated with increased likelihoods of mild cognitive impairment and non-amnestic mild cognitive impairment and lower scores in global cognition, verbal fluency, attention and executive function (Bonferroni-corrected *P* < 0.05). In the subsamples, long sleep duration was associated with higher plasma amyloid-β40 and total tau, a lower amyloid-β42/amyloid-β40 ratio and smaller grey matter volume (Bonferroni-corrected *P* < 0.05). Sleep duration was not significantly associated with serum-soluble adhesion molecules, white matter hyperintensity volume, global enlarged perivascular spaces and lacunes (*P* > 0.05). Alzheimer’s and neurodegenerative pathologies may represent common pathways linking long sleep duration with mild cognitive impairment and low cognition in older adults.

## Teaser

Association of Objective Sleep Duration with Cognition and Brain Aging Biomarkers in Older Adults. Tang *et al.* revealed that objective long sleep duration was associated with mild cognitive impairment, low cognition and biomarkers for Alzheimer’s disease and neurodegeneration but not with biomarkers for microvascular dysfunction in dementia-free older adults, suggesting that neurodegenerative pathologies may be involved in both long sleep duration and cognitive impairment.

## Graphical Abstract

**Graphical Abstract:**
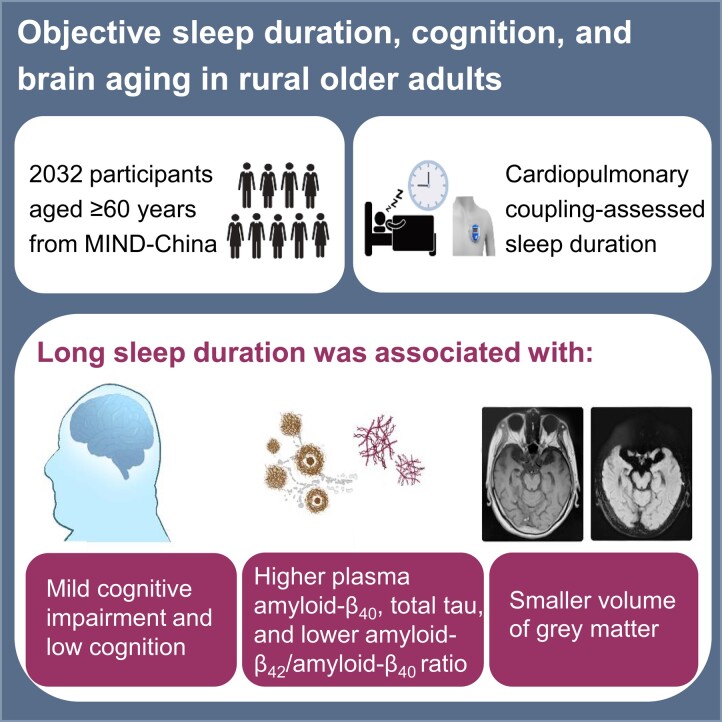

## Introduction

Mild cognitive impairment (MCI), as an intermediate condition between normal cognitive aging and dementia, affects around 20–25% of people aged 60 years or above.^1,2^ People with MCI are at an increased risk for progressing to dementia.^3^ Mounting evidence has suggested that sleep disturbances, such as long and short sleep duration, are associated with accelerated cognitive decline in older adults.^4-6^ A meta-analysis of case-control studies suggested that patients with MCI experienced more serious sleep disturbances (e.g. self-reported longer sleep duration) and that the patterns of sleep disturbances might differ by MCI subtypes.^7^ However, a few studies that examine self-reported sleep duration in association with MCI and its progression to dementia have yielded mixed results,^8,9^ which might be partly attributable to bias of self-reported sleep information. In addition, the relationships between objectively measured sleep duration with subtypes of MCI have rarely been investigated among community-dwelling older adults.

The mechanisms underlying the association between abnormal sleep duration and low cognitive function remain poorly understood. The PET imaging or CSF studies have linked sleep deprivation or insufficient sleep duration with an increased burden of amyloid-β (Aβ) and neurodegeneration in central nervous system among older adults.^4,10^ In addition, a memory clinic-based study showed that self-reported short sleep duration (<6 versus > 7 h) was associated with high plasma Aβ42 among dementia-free older adults.^11^ To date, however, the associations of objective sleep duration with peripheral biomarkers for Alzheimer’s (e.g. Aβ), neurodegeneration [e.g. total tau (t-tau)^12^ and neurofilament light chain (NfL) proteins] and microvascular dysfunction (e.g. soluble adhesion molecules) have not been examined in the general population settings.

Furthermore, autopsy-verified MRI studies reveal that global and regional brain atrophy (e.g. grey matter and hippocampus) are reliable imaging markers for the extent of neurodegeneration due to neurofibrillary tangles and neuronal loss,^13,14^ whereas lacunes, perivascular spaces and white matter hyperintensity (WMH) largely reflect cerebral microvascular lesions.^15^ Previous studies suggested that grey matter and hippocampal atrophy were correlated with self-reported long (e.g. > 7 h) or short (e.g. < 6 h) sleep duration in cognitively unimpaired older adults,^16-19^ suggesting that both long and short sleep duration may be an early sign of neurodegeneration. However, it remains unclear whether long or short sleep duration is associated with cerebral microvascular lesions. Taken together, exploring the associations of objectively measured sleep duration with multimodal biomarkers of microvascular lesions, Alzheimer’s disease and neurodegeneration may shed light on the neuropathological mechanisms underlying the association of sleep duration with cognitive phenotypes.

Therefore, in this population-based study of rural-dwelling Chinese older adults, we sought to (i) examine the associations between objectively measured sleep duration with MCI, subtypes of MCI and performance in various cognitive domains and (ii) further evaluate the relationships of sleep duration with blood and MRI biomarkers of amyloid pathology, neurodegeneration and microvascular lesions. We hypothesize that long or short sleep duration is associated with MCI and low cognitive performance in older adults and that certain common neuropathologies may underlie the associations.

## Materials and methods

### Study design and participants

This population-based study used data derived from the Multimodal Interventions to Delay Dementia and Disability in Rural China (MIND-China) study, which was part of the World-Wide FINGERS Network.^20^ The comprehensive baseline assessments of MIND-China were reported in detail elsewhere.^21-23^ Briefly, the baseline examinations of MIND-China engaged rural residents who were aged ≥60 years and living in 52 villages from Yanlou Town, western Shandong Province. In March–September 2018, 5765 participants (74.9% of all eligible people) were examined for MIND-China. In July 2018–November 2019, a village-based subsample (*n* = 2340) who were free from arrhythmia derived from MIND-China participants undertook the cardiopulmonary coupling (CPC) substudy. Of these, 308 persons were excluded due to suboptimal quality of CPC data (*n* = 196), prevalent dementia (*n* = 50), major mental disorders (*n* = 13) and missing data on 4 cognitive domains (*n* = 49). Thus, the analytical sample included 2032 participants. Of these, data were available in 841 participants for Alzheimer’s disease–related plasma biomarkers, in 1044 for peripheral biomarkers of microvascular dysfunction and in 834 for structural brain MRI markers. Supplementary Fig. 1 shows the flowchart of the study participants.

The Ethics Committee at the Shandong Provincial Hospital in Jinan approved the MIND-China protocol. All participants or informants provided written informed consent prior to the examinations. MIND-China was formally registered in the Chinese Clinical Trial Registry (registration no.: ChiCTR1800017758).

### Data collection and definitions

In March–September 2018, the trained medical staff collected data through in-person interviews, clinical examinations, psychological testing and laboratory tests. Data collection, definitions, assessments and categorizations of covariates are described in detail in Supplementary Text 1.

### Assessments of sleep characteristics

In the MIND-China CPC substudy, we used the electronic electrocardiogram (ECG)-based CPC technique to measure sleep duration and wake after sleep onset (WASO). In brief, all eligible participants were instructed to wear the CPC monitor (AECG-600D, Nanjing Fengshengyongkang Software Technology Co., Ltd., Jiangsu, China) and go to sleep at their usual bedtimes at home for 24 h, as previously reported.^24,25^ The CPC method used a continuous single-channel ECG to analyse heart rate variability and the ECG-derived respiration signal. The CPC data were uploaded to the SleepImage^TM^ website, and then, the automatic analysis generated CPC variables, and the sleep spectrogram graphed total sleep time and WASO (actigraphy). The detailed description of the original methodology of the CPC algorithm has been previously reported.^25,26^ We categorized nocturnal sleep duration (h) into short (≤6), normal (6–8, reference) and long (>8) duration based on previous studies.^27,28^ This categorization was also supported by the restricted cubic spline analysis, which showed statistically marginal J-shaped associations of the CPC-measured sleep duration with MCI (*P* for non-linear = 0.100) (Supplementary Fig. 2).

### Assessments of cognitive function and diagnosis of MCI and subtypes

We used a neuropsychological test battery to assess cognitive function, as previously reported^2^ and fully described in the Supplementary Text 1 as well. In brief, we assessed function of four cognitive domains:^2^ memory, language, attention and executive function. The raw cognitive test scores were first standardized into *Z*-scores and standard deviations (SD), derived from all dementia-free participants in the MIND-China study. Then, because all four cognitive domains were assessed using more than one test, we created the composite *Z*-score for each cognitive domain by averaging the *Z*-scores of all tests for that domain. A composite global cognitive *Z*-score was computed as the mean of all *Z*-scores for individuals with data available in at least two of the four cognitive domains. Objective cognitive impairment was defined as scoring ≥1.0 SD below the age- and education-specific mean among dementia-free participants in MIND-China in any of the four cognitive domains. MCI and subtypes of MCI were defined following the Petersen’s criteria that were operationalized following an approach similar to that used in the Mayo Clinic Study of Aging, as previously described.^2,29^ The final judgment about MCI was made according to both psychological test scores and a consensus agreement among neurologists specialized in clinical diagnosis and care of dementia disorders. We further classified MCI into amnestic MCI (aMCI) if the memory domain was impaired or non-amnestic MCI (naMCI) if there was no impairment in memory function.

### Measurements of blood biomarkers for Alzheimer’s disease, neurodegeneration and microvascular lesions

After an overnight fast, blood samples were collected into ethylenediaminetetra-acetic acid (EDTA)-coated tubes. EDTA plasma and serum samples were aliquoted and stored at −80°C according to standard procedures until further analysis. Plasma Aβ40, Aβ42, t-tau and NfL were measured on a single molecule array (Simoa) platform (Quanterix Corp, MA, USA) following the manufacturer’s instructions (Wayen Biotechnologies Inc., Shanghai, China).^30^ We used the Human Neurology 3-Plex A assay to measure plasma Aβ and t-tau and the NF-light® Advantage Kit for plasma NfL. We used ‘Vascular Injury Panel 2 (human)’ commercial kit from Meso Scale Discovery to quantify serum cytokine concentrations. The concentrations of serum-soluble intercellular adhesion molecule 1 (ICAM-1) and vascular cell adhesion molecule 1 (VCAM-1) were assayed following the manufacturer’s instructions. The quality control procedure was described elsewhere.^31^

### Acquisition and processing of MRI data

Participants were scanned on either the Philips Ingenia 3.0 T MR scanner (Philips Healthcare, Best, The Netherlands) in Shandong Southwestern Lu Hospital (*n* = 750) or the Philips Archiva 3.0 T MR scanner (Philips Healthcare, Best, The Netherlands) in Liaocheng People’s Hospital (*n* = 84). The parameters of the core MRI sequences were fully reported elsewhere,^21^ and the image processing and assessments of brain MRI markers were provided in detail in Supplementary Text 1.

### Statistical analysis

The characteristics of study participants by cognitive status were compared using *t*-test for normally distributed continuous variables, Mann–Whitney *U* test for skewed-distributed continuous variables and χ^2^ test for categorical variables. The non-linear association of MCI with sleep duration and WASO was assessed using restricted cubic spline curve analysis, in which binary logistic regression models were used with three knots at the 10th, 50th and 90th percentiles of sleep duration. We used binary logistic regression models to estimate the odds ratio (OR) and 95% confidence interval (CI) of MCI associated with sleep duration and multinomial logistic regression models to examine the associations of sleep duration with aMCI and naMCI.

General linear regression models were used to estimate the relationship of sleep duration with cognitive *Z*-scores, blood biomarkers and volumetric brain MRI measures. We employed binary logistic regression models to estimate the OR (95% CI) of lacunes associated with sleep duration. Plasma Aβ40 and NfL data were log-transformed to reduce skewness. We presented main results from two models: Model 1 was adjusted for age, sex, education and, wherever applicable, for MRI scan centres and intracranial volume (ICV). Model 2 was additionally adjusted for lifestyle factors (e.g. alcohol consumption and smoking), clinical conditions (e.g. diabetes, coronary heart disease and stroke), depressive symptoms, use of hypnotics and apolipoprotein E (*APOE*) genotype. We created a dummy variable to represent people with missing values for a categorical co-variable, and missing values of a continuous co-variable were replaced with mean values to maximize the analytical sample. We tested the statistical interactions of sleep duration with age (<75 versus ≥ 75 years), sex or *APOE* ɛ4 allele (carriers versus non-carriers) on the likelihood of MCI and cognitive *Z*-scores by simultaneously entering the independent variables and their cross-product term into the model. Stratified analyses were performed when a statistical interaction was detected at *P* < 0.05. Finally, in the sensitivity analyses, to examine whether sleep duration was associated with function of subcognitive domains among people with normal cognitive function, we repeated the analyses by excluding individuals with MCI from the analytical sample.

The R Statistical Software for Windows (version 4.2.0, R Foundation for Statistical Computing, Vienna, Austria) was used for all statistical analyses. A two-tailed *P* < 0.05 after the Bonferroni correction for multiple comparisons was considered statistically significant.

## Results

### Characteristics of study participants

Out of the 2032 participants, the mean age was 69.56 (SD = 4.54) years, 55.1% were women, and 34.6% did not attend formal schooling. Of these, MCI was defined in 510 persons (25.1%), including 438 with aMCI and 72 with naMCI. Individuals with MCI (versus normal cognition) were older, less educated, more likely to be females and underweight, less likely to smoke and consume alcohol and more likely to have hypertension, stroke and depressive symptoms and had lower *Z*-scores of the four examined cognitive domains and global cognition (*P* < 0.05) (Table 1). There were no significant differences between the two groups in the distribution of diabetes, use of hypnotics, coronary heart disease, *APOE* ε4 allele and CPC-measured sleep duration (*P* > 0.05). In the subsamples of plasma biomarkers (*n* = 841, 214 persons with MCI; of these, 189 with aMCI and 25 with naMCI), serum adhesion molecules (*n* = 1044, 268 persons with MCI; of these, 237 with aMCI and 31 with naMCI) and structural brain MRI biomarkers (*n* = 834, 183 persons with MCI; of these, 166 with aMCI and 17 with naMCI), individuals with MCI had higher plasma Aβ40; higher serum adhesion molecules ICAM-1 and VCAM-1; smaller volumes of the total brain tissue, total grey matter, total white matter and hippocampus; and higher global WMH volume (*P* < 0.05) (Table 1).

**Table 1:** Characteristics of the study participants by mild cognitive impairment (*n* = 2032)

Characteristics^a^	Total sample (*n* = 2032)	Mild cognitive impairment		
		No (*n* = 1522)	Yes (*n* = 510)	*P-*value
Age (years)	69.56 (4.54)	69.26 (4.49)	70.45 (4.55)	<0.001
Female, *n* (%)	1119 (55.1)	783 (51.4)	336 (65.9)	<0.001
Educational level, *n* (%)				<0.001
Illiteracy	704 (34.6)	460 (30.2)	244 (47.8)	
Elementary school	895 (44.0)	674 (44.3)	221 (43.3)	
Middle school or above	433 (21.3)	388 (25.5)	45 (8.8)	
Body mass index (kg/m^2^)	25.02 (3.59)	25.15 (3.59)	24.66 (3.56)	0.023
Alcohol drinking, *n* (%)				<0.001
Never	1167 (57.4)	814 (53.5)	353 (69.2)	
Former	178 (8.8)	138 (9.1)	40 (7.8)	
Current	687 (33.8)	570 (37.5)	117 (22.9)	
Smoking, *n* (%)				<0.001
Never	1256 (61.8)	893 (58.7)	363 (71.2)	
Former	471 (23.2)	378 (24.8)	93 (18.2)	
Current	305 (15.0)	251 (16.5)	54 (10.6)	
Hypertension, *n* (%)	1333 (66.2)	978 (64.9)	355 (69.7)	0.048
Diabetes, *n* (%)	304 (15.0)	235 (15.4)	69 (13.5)	0.295
Coronary heart disease, *n* (%)	452 (22.2)	328 (21.6)	124 (24.3)	0.194
Stroke, *n* (%)	293 (14.4)	202 (13.3)	91 (17.8)	0.011
Depressive symptoms, *n* (%)	159 (7.9)	90 (6.0)	69 (13.7)	<0.001
Hypnotics use, *n* (%)	90 (4.5)	63 (4.2)	27 (5.4)	0.256
*APOE* ε4 allele carrier, *n* (%)	319 (16.0)	237 (15.9)	82 (16.4)	0.786
Sleep parameters				
CPC-measured sleep duration (h)	7.33 (1.51)	7.29 (1.48)	7.43 (1.59)	0.085
Wake after sleep onset (h)	1.21 (0.71)	1.22 (0.71)	1.17 (0.72)	0.061
Cognitive domains^b^, *Z*-score				
Global cognition	0.03 (0.66)	0.25 (0.56)	−0.65 (0.44)	<0.001
Memory	0.05 (0.87)	0.35 (0.70)	−0.86 (0.68)	<0.001
Verbal fluency	0.07 (0.80)	0.27 (0.73)	−0.54 (0.69)	<0.001
Attention	0.04 (0.86)	0.22 (0.81)	−0.51 (0.75)	<0.001
Executive function	−0.03 (0.94)	0.18 (0.85)	−0.69 (0.90)	<0.001
Plasma biomarkers (*n* = 841)				
Aβ42 (pg/mL)	11.83 (2.93)	11.79 (2.93)	11.96 (2.90)	0.550
Aβ40 (pg/mL)	176.10 (45.96)	174.32 (46.12)	181.32 (45.21)	0.031
Aβ42/Aβ40 ratio^c^	69.08 (16.20)	69.57 (16.43)	67.65 (15.44)	0.093
Total tau (pg/mL)	2.36 (0.96)	2.36 (0.95)	2.36 (1.01)	0.688
NfL (pg/mL)	14.95 (20.32)	14.49 (17.25)	16.28 (27.41)	0.079
Serum adhesion molecules (*n* = 1044)				
ICAM-1 (ng/mL)	486.71 (124.68)	477.31 (109.95)	513.92 (156.94)	<0.001
VCAM-1 (ng/mL)	632.38 (146.60)	623.94 (129.68)	656.82 (185.32)	0.009
Brain MRI markers (*n* = 834)				
Intracranial volume (cm^3^)	1364.47 (145.34)	1369.32 (144.97)	1347.23 (145.73)	0.053
Total brain tissue volume (cm^3^)	982.00 (97.14)	987.31 (97.00)	963.13 (95.54)	0.003
Grey matter volume (cm^3^)	526.72 (51.65)	529.52 (51.50)	516.76 (51.11)	0.003
White matter volume (cm^3^)	450.56 (52.41)	453.22 (52.54)	441.09 (51.00)	0.011
Hippocampus volume (cm^3^)	5.64 (0.61)	5.67 (0.60)	5.52 (0.63)	0.003
Total WMH volume (cm^3^)	8.26 (10.64)	7.91 (10.63)	9.52 (10.58)	0.011
No. of EPVS	63.58 (28.90)	63.09 (28.72)	65.32 (29.53)	0.359
Lacune, *n* (%)	218 (26.1)	163 (25.0)	55 (30.1)	0.172

Data are mean (standard deviation), unless otherwise specified.

*APOE*, apolipoprotein E gene; CPC, cardiopulmonary coupling; EPVS, enlarged perivascular spaces; ICAM-1, intercellular adhesion molecule 1; MRI, magnetic resonance imaging; NfL, neurofilament light; VCAM-1, vascular cellular adhesion molecule 1; WMH, white matter hyperintensity.

^a^Numbers of participants with missing values are 10 for body mass index, 17 for hypertension, 20 for depressive symptoms, 11 for use of hypnotics and 44 for *APOE* genotype. In subsequent analyses, categorical variable with missing values was replaced with a dummy variable and continuous variable with missing values was replaced with a mean value.

^b^Numbers of participants with missing values are 6 for global cognitive *Z*-score, 24 for memory *Z*-score, 11 for verbal fluency *Z*-score, 14 for attention *Z*-score and 28 for executive function *Z*-score.

^c^Plasma Aβ42/Aβ40 ratio was calculated and multiplied by 1000.

### Associations of sleep duration with MCI, aMCI and naMCI (*n* = 2032)

Controlling for age, sex and education, having objective sleep duration >8 h (versus 6–8 h) was significantly associated with increased likelihoods of MCI and naMCI (*P* < 0.05 after the Bonferroni correction), and these associations remained statistically significant in model 2 when additionally controlling for *APOE* ε4 allele, lifestyle factors and metabolic and clinical factors (Table 2). After the Bonferroni correction, the association of long sleep duration (>8 versus 6–8 h) with aMCI became non-significant (Table 2). Short sleep duration (≤6 versus 6–8 h) was not significantly associated with MCI, aMCI and naMCI (*P* > 0.05 after the Bonferroni correction). In addition, there were no statistical interactions of sleep duration with age groups, sex and *APOE* ε4 allele on the likelihoods of MCI and subtypes (*P* for all interactions > 0.05). The restrict cubic spline analysis showed no significant associations of MCI with the CPC-measured WASO (Supplementary Fig. 3).

**Table 2:** Associations between CPC-measured sleep duration and mild cognitive impairment and subtypes of mild cognitive impairment (*n* = 2032)

Sleep duration	No. of subjects	No. of cases	Odds ratio (95% CI), MCI or subtypes of MCI			
			Model 1^a^	*P*-value	Model 2^a^	*P*-value
MCI						
Sleep duration						
≤6 h	386	94	1.06 (0.8–1.41)	0.702	1.05 (0.79–1.41)	0.725
6–8 h	996	224	1.00 (reference)		1.00 (reference)	
>8 h	650	192	1.44 (1.14–1.82)	0.002*	1.41 (1.11–1.78)	0.005*
Amnestic MCI						
Sleep duration						
≤6 h	386	83	1.07 (0.79–1.44)	0.658	1.06 (0.78–1.43)	0.726
6–8 h	996	197	1.00 (reference)		1.00 (reference)	
>8 h	650	158	1.34 (1.04–1.72)	0.021	1.31 (1.02–1.69)	0.034
Non-amnestic MCI						
Sleep duration						
≤6 h	386	11	0.98 (0.48–2.02)	0.966	1.06 (0.51–2.22)	0.877
6–8 h	996	27	1.00 (reference)		1.00 (reference)	
>8 h	650	34	2.26 (1.33–3.82)	0.003*	2.12 (1.23–3.67)	0.007*

CI, confidence interval; CPC, cardiopulmonary coupling; MCI, mild cognitive impairment.

^a^Model 1 was adjusted for age, sex and education. Model 2 was additionally adjusted for body mass index, alcohol consumption, smoking, hypertension, diabetes, coronary heart disease, stroke, depressive symptoms, use of hypnotics and *APOE* genotype.

^*^ *P* < 0.05 after the Bonferroni correction for multiple comparison tests.

### Associations of sleep duration with cognitive function (*n* = 2032)

Long sleep duration (>8 versus 6–8 h) was significantly associated with a reduced global cognitive *Z*-score and decreased *Z*-scores of verbal fluency, attention and executive function domains, even when adjusting for multiple potential confounding variables (*P* < 0.05 after the Bonferroni correction) (Table 3). In addition, there were no significant associations of short sleep duration (≤6 versus 6–8 h) with various cognitive domains (*P* > 0.05 after the Bonferroni correction) (Table 3). Short sleep duration was significantly associated with a reduced composite *Z*-score of verbal fluency, but the association became non-significant after Bonferroni correction (Table 3). There were no statistical interactions of sleep duration with age groups, sex and *APOE* ε4 allele on any of the examined subcognitive domains (*P* for all interactions > 0.05) (Table 3).

**Table 3:** Associations between CPC-measured sleep duration and cognitive function among dementia-free participants (*n* = 2032)

Sleep duration	No. of participants	β coefficient (95% CI), cognitive *Z*-score			
		Model 1^a^	*P*-value	Model 2^a^	*P*-value
Global cognition^b^					
Sleep duration					
≤6 h	385	−0.04 (−0.10 to 0.02)	0.226	−0.04 (−0.10 to 0.03)	0.253
6 to 8 h	994	0.00 (reference)		0.00 (reference)	
>8 h	647	−0.12 (−0.17 to −0.07)	0.001*	−0.11 (−0.17 to −0.06)	<0.001*
Memory^b^					
Sleep duration					
≤6 h	385	−0.01 (−0.11 to 0.09)	0.866	−0.004 (−0.10 to 0.09)	0.929
6 to 8 h	983	0.00 (reference)		0.00 (reference)	
>8 h	640	−0.09 (−0.17 to −0.01)	0.029	−0.08 (−0.16 to 0.002)	0.056
Verbal fluency^b^					
Sleep duration					
≤6 h	385	−0.09 (−0.18 to −0.002)	0.046	−0.09 (−0.17 to −0.0001)	0.050
6 to 8 h	991	0.00 (reference)		0.00 (reference)	
>8 h	645	−0.14 (−0.21 to −0.06)	<0.001*	−0.13 (−0.20 to −0.06)	<0.001*
Attention^b^					
Sleep duration					
≤6 h	385	−0.05 (−0.14 to 0.03)	0.233	−0.05 (−0.13 to 0.04)	0.285
6 to 8 h	990	0.00 (reference)		0.00 (reference)	
>8 h	643	−0.13 (−0.20 to −0.06)	<0.001*	−0.12 (−0.19 to −0.05)	0.001*
Executive function^b^					
Sleep duration					
≤6 h	384	−0.01 (−0.10 to 0.08)	0.808	−0.01 (−0.10 to 0.08)	0.866
6 to 8 h	984	0.00 (reference)		0.00 (reference)	
>8 h	636	−0.12 (−0.20 to −0.04)	0.002*	−0.12 (−0.19 to −0.04)	0.004*

CI, confidence interval; CPC, cardiopulmonary coupling.

^a^Model 1 was adjusted for age, sex and education. Model 2 was additionally adjusted for body mass index, alcohol consumption, smoking, hypertension, diabetes, coronary heart disease, stroke, depressive symptoms, use of hypnotics and *APOE* genotype.

^b^Numbers of participants with missing values are 6 for global cognitive *Z*-score, 24 for memory *Z*-score, 11 for verbal fluency *Z*-score, 14 for attention *Z*-score and 28 for executive function *Z*-score.

^*^ *P* < 0.05 after the Bonferroni correction for multiple comparison tests.

### Sleep duration and peripheral biomarkers

In the subsample of plasma biomarkers (*n* = 841), long sleep duration (>8 versus 6–8 h) was significantly associated with higher concentrations of plasma Aβ40 and t-tau and a lower Aβ42/Aβ40 ratio but not with plasma Aβ42 and NfL after controlling for demographic factors (*P* for all linear trends < 0.05 after the Bonferroni correction) and these associations remained largely the same in the fully adjusted models (Table 4). Short sleep duration (≤6 versus 6–8 h) was not significantly associated with plasma biomarkers for Alzheimer’s disease and neurodegeneration (*P* > 0.05 after the Bonferroni correction). Long sleep duration and short sleep duration was significantly associated with higher plasma NfL in the multivariable-adjusted models, but the associations became non-significant after Bonferroni correction (Table 4). In the subsample of serum biomarkers for microvascular dysfunction (*n* = 1044), sleep duration was not significantly associated with serum adhesion molecules ICAM-1 or VCAM-1 (*P* > 0.05) (Table 4).

**Table 4:** Associations of CPC-measured sleep duration with plasma biomarkers and serum adhesion molecules among dementia-free participants

Sleep duration	No. of participants	β coefficient (95% CI), plasma biomarkers			
		Model 1^a^	*P*-value	Model 2^a^	*P*-value
Plasma biomarkers (*n* = 841)					
Aβ42 (pg/mL)					
Sleep duration					
≤6 h	161	0.16 (−0.38 to 0.69)	0.570	0.26 (−0.27 to 0.79)	0.340
6 to 8 h	422	0.00 (reference)		0.00 (reference)	
>8 h	258	0.03 (−0.42 to 0.49)	0.883	−0.03 (−0.49 to 0.42)	0.885
Aβ40 (pg/ml)^b^					
Sleep duration					
≤6 h	161	0.01 (−0.04 to 0.05)	0.735	0.01 (−0.03 to 0.06)	0.572
6 to 8 h	422	0.00 (reference)		0.00 (reference)	
>8 h	258	0.07 (0.03 to 0.11)	<0.001*	0.07 (0.03 to 0.10)	<0.001*
Aβ42/Aβ40 ratio^c^					
Sleep duration					
≤6 h	161	0.17 (−2.77 to 3.11)	0.908	0.41 (−2.54 to 3.35)	0.786
6 to 8 h	422	0.00 (reference)		0.00 (reference)	
>8 h	258	−4.90 (−7.41 to −2.40)	<0.001*	−4.85 (−7.35 to −2.34)	<0.001*
Total tau (pg/mL)					
Sleep duration					
≤6 h	161	0.01 (−0.16 to 0.19)	0.868	−0.003 (−0.18 −0.17)	0.977
6 to 8 h	422	0.00 (reference)		0.00 (reference)	
>8 h	258	0.23 (0.08 to 0.38)	0.003*	0.23 (0.08 to 0.38)	0.003*
NfL (pg/mL)^b^					
Sleep duration					
≤6 h	161	0.08 (−0.01 to 0.16)	0.070	0.09 (0.004 to 0.17)	0.041
6 to 8 h	422	0.00 (reference)		0.00 (reference)	
>8 h	258	0.09 (0.02 to 0.16)	0.015	0.07 (0.003 to 0.14)	0.040
Serum adhesion molecules (*n* = 1044)					
ICAM-1 (ng/mL)					
Sleep duration					
≤6 h	195	14.87 (−5.86 to 35.60)	0.160	13.10 (−7.84 to 34.04)	0.220
6 to 8 h	515	0.00 (reference)		0.00 (reference)	
>8 h	334	12.17 (−5.05 to 29.40)	0.166	12.20 (−5.23 to 29.63)	0.170
VCAM-1 (ng/mL)					
Sleep duration					
≤6 h	195	2.61 (−21.69 to 26.91)	0.833	3.44 (−21.02 to 27.90)	0.783
6 to 8 h	515	0.00 (reference)		0.00 (reference)	
>8 h	334	13.13 (−7.05 to 33.32)	0.202	12.41 (−7.95 to 32.77)	0.232

Aβ, amyloid beta; CI, confidence interval; CPC, cardiopulmonary coupling; ICAM-1, intercellular adhesion molecule 1; NfL, neurofilament light chain; VCAM-1, vascular cellular adhesion molecule 1.

^a^Model 1 was adjusted for age, sex and education. Model 2 was additionally adjusted for body mass index, alcohol consumption, smoking, hypertension, diabetes, coronary heart disease, stroke, depressive symptoms, use of hypnotics and *APOE* genotype.

^b^The original data of the biomarkers were natural log-transformed due to skewed distributions.

^c^Plasma Aβ42/Aβ40 ratio was calculated and multiplied by 1000.

^*^ *P* < 0.05 after the Bonferroni correction for multiple comparison tests.

### Sleep duration and structural brain MRI markers (*n* = 834)

In the subsample of brain MRI biomarkers (*n* = 834), long sleep duration (>8 versus 6–8 h) was significantly associated with smaller volumes of grey matter, after controlling for demographic factors (*P* < 0.05 after the Bonferroni correction) (Table 5). These associations remained statistically significant in the fully adjusted models. Following the Bonferroni correction, the associations of long sleep duration with reduced total brain tissue volume and hippocampal volume became statistically non-significant (Table 5). Objective sleep duration was not significantly associated with white matter and WMH volumes, global enlarged perivascular spaces (EPVS) and lacunes (*P* > 0.05 after the Bonferroni correction) (Table 5).

**Table 5:** Associations between CPC-measured sleep duration and brain MRI markers among dementia-free participants (*n* = 834)

Sleep duration	No. of participants	β coefficient or odds ratio (95% CI), brain MRI measures			
		Model 1^a^	*P*-value	Model 2^a^	*P*-value
Total brain tissue volume (cm^3^)					
Sleep duration					
≤6 h	165	−3.95 (−14.11 to 6.22)	0.446	−4.05 (−14.28 to 6.18)	0.438
6 to 8 h	421	0.00 (reference)		0.00 (reference)	
>8 h	248	−10.53 (−19.32 to −1.74)	0.019	−9.83 (−18.68 to −0.99)	0.029
Grey matter volume (cm^3^)					
Sleep duration					
≤6 h	165	−2.23 (−7.93 to 3.48)	0.444	−2.30 (−8.06 to 3.46)	0.433
6 to 8 h	421	0.00 (reference)		0.00 (reference)	
>8 h	248	−8.27 (−13.20 to −3.34)	0.001*	−8.24 (−13.22 to −3.26)	0.001*
White matter volume (cm^3^)					
Sleep duration					
≤6 h	165	−1.73 (−8.04 to 4.59)	0.592	−2.01 (−8.35 to 4.34)	0.535
6 to 8 h	421	0.00 (reference)		0.00 (reference)	
>8 h	248	−2.62 (−8.09 to 2.84)	0.346	−1.88 (−7.37 to 3.60)	0.501
Hippocampal volume (cm^3^)					
Sleep duration					
≤6 h	165	−0.06 (−0.16 to 0.04)	0.261	−0.05 (−0.15 to 0.05)	0.345
6 to 8 h	421	0.00 (reference)		0.00 (reference)	
>8 h	248	−0.12 (−0.21 to −0.03)	0.007	−0.11 (−0.20 to −0.03)	0.011
WMH volume (cm^3^)^b^					
Sleep duration					
≤6 h	165	0.01 (−0.11 to 0.13)	0.859	0.04 (−0.08 to 0.16)	0.543
6 to 8 h	421	0.00 (reference)		0.00 (reference)	
>8 h	248	0.05 (−0.06 to 0.15)	0.370	0.04 (−0.07 to 0.14)	0.481
EPVS					
Sleep duration					
≤6 h	165	−4.67 (−9.75 to 0.40)	0.071	−4.24 (−9.34 to 0.86)	0.103
6 to 8 h	421	0.00 (reference)		0.00 (reference)	
>8 h	248	−1.08 (−5.47 to 3.31)	0.629	−0.83 (−5.24 to 3.58)	0.712
Lacunes					
Sleep duration					
≤6 h	165	0.67 (0.43 to 1.05)	0.080	0.72 (0.45 to 1.15)	0.172
6 to 8 h	421	1.00 (reference)		1.00 (reference)	
>8 h	248	0.92 (0.65 to 1.32)	0.654	0.84 (0.58 to 1.22)	0.355

CI, confidence interval; CPC, cardiopulmonary coupling; EPVS, enlarged perivascular spaces; MRI, magnetic resonance imaging; WMH, white matter hyperintensity.

^a^Model 1 was adjusted for age, sex, education, MRI scan centres and total intracranial volume for all volumetric measures. Model 2 was additionally adjusted for body mass index, alcohol consumption, smoking, hypertension, diabetes, coronary heart disease, stroke, depressive symptoms, use of hypnotics and *APOE* genotype.

^b^WMH volume was cubic root transformed to normalize the distributions.

^*^ *P* < 0.05 after the Bonferroni correction for multiple comparison tests.

### Sensitivity analysis

To examine whether objective sleep duration was associated with various cognitive domains among cognitively normal individuals, we repeated the analyses among individuals free of MCI (*n* = 1522). The results showed that long sleep duration (>8 versus 6–8 h) was only significantly associated with a reduced composite *Z*-score of global cognition (*P* < 0.05 after the Bonferroni correction) (Supplementary Table 1).

## Discussion

In this population-based cross-sectional study of rural-dwelling dementia-free older adults in China, the main findings can be summarized as follows: (i) objectively measured long sleep duration (>8 versus 6–8 h) was associated with increased likelihoods of MCI and naMCI as well as low verbal fluency, attention, executive function and global cognitive function, independent of sociodemographic, genetic, behavioural and clinical factors, and (2) long sleep duration was correlated with peripheral and neuroimaging biomarkers for Alzheimer’s disease and neurodegeneration (e.g. higher plasma Aβ40 and t-tau, lower Aβ42/Aβ40 ratio and lower grey matter volume) but not for cerebral microvascular lesions or dysfunction (e.g. serum ICAM-1 and VCAM-1, WMH volume, EPVS and lacunes). Taken together, these results imply that long sleep duration can be a useful clinical marker for prodromal dementia in older adults and that long sleep duration and MCI may share common Alzheimer’s disease pathology and neurodegeneration.

Previously, population-based studies of elderly people have frequently linked self-reported long sleep duration with MCI;^32^ most of these studies had been conducted among urban populations. This implies that long sleep duration may represent a clinical marker that occurs already at the prodromal phase of the dementia syndrome. Our community-based study further revealed that objectively measured long sleep duration was independently associated with MCI, especially naMCI, which was in good agreement with the previous systematic review.^7^ The differences in sleep disturbances between aMCI and naMCI could be partly attributable to different neuropathologies underlying subtypes of MCI.^33^

Our study showed the association between long sleep duration and low cognitive function in multiple cognitive domains except memory, which is consistent with the report from the multicentre cross-sectional study of community-dwelling older men in the USA.^34^ In addition, a meta-analysis of population-based studies showed that not only long sleep duration but also self-reported short sleep duration (e.g. < 4 h) was associated with poor performance in global cognitive function and multiple cognitive domains such as executive function, verbal fluency and working memory,^6^ whereas we found an overall J-shaped relationship of objective sleep duration with cognitive performance where the association of objective short sleep duration (<6 h) with poor cognitive function was not statistically evident. This meta-analysis included population-based studies mainly from the USA and Northern Europe, and studies of middle-aged and older adults have suggested that sleep characteristics (e.g. time in bed, sleep duration and daytime sleepiness) differ between rural and urban residents and across ethnic groups.^22,35^ Thus, the slight discrepant findings across studies might be partially owing to differences in genetic, cultural, environmental, lifestyle and psychosocial factors among different study populations.^36,37^ Future prospective cohort studies are needed to compare the relationship between objective sleep duration and cognitive function among different ethnic populations.

Our population-based study of rural residents revealed for the first time the association of objective long sleep duration with a lower plasma Aβ42/Aβ40 ratio, in line with the report from a clinical-based study in China that showed an association between long sleep duration and lower CSF Aβ42/Aβ40 ratio.^38^ A lower plasma Aβ42/40 ratio has been correlated with a higher load of cerebral Alzheimer’s disease pathology.^39^ Thus, current evidence from our study and the literature tends to support the view that abnormal sleep duration is associated with biomarkers of brain Aβ burden in older adults.

We also detected an association of objective long sleep duration with higher plasma t-tau, a biomarker for neurodegeneration.^39,40^ This is in contrast with the report from the Rotterdam Study, where no association between objectively measured sleep duration with plasma t-tau was found.^41^ The discrepant finding might be partly due to the fact that the Rotterdam Study sample was relatively younger than our sample (mean age, 66.7 versus 69.6 years) and that plasma concentrations of t-tau increase with age.^31^ The associations of abnormal sleep duration with multimodal biomarkers for Alzheimer’s disease and neurodegenerative pathology among dementia-free older adults deserve further investigation in prospective cohort studies.

Previous studies have not fully characterized structural brain alterations associated with objective sleep duration in older adults. The association between long sleep duration and low cognitive function may be partly attributable to early neurodegenerative pathology in the brain. In support of this view, our study did show associations of long sleep duration with reduced total grey matter volume. This is consistent with report from the Framingham Heart Study, which demonstrated that prolonged sleep duration was an early clinical marker of neurodegeneration.^42^ The mechanisms underpinning long sleep duration in older adults may extend to neurodegenerative pathologies in certain brain regions that are involved in the regulation of the cycle of sleep and wakefulness. This may lead to circadian dysfunction, misalignment and altered production and secretion of melatonin^43^ Of note, we found no evidence supporting the association of sleep duration with peripheral and structural brain MRI biomarkers for microvascular lesions (e.g. serum adhesion molecules ICAM-1 and VCAM-1, WMHs, EPVS and lacunes). The lack of association between sleep duration and MRI markers of cerebral microvascular lesions was overall consistent with reports from the Lothian Birth Cohort 1936 study (e.g. WMHs and EPVS) and the Rotterdam Study (e.g. EPVS).^44,45^ Taken together, alterations in sleep duration among older adults may begin in the preclinical stage of dementia and could be a potential clinical marker for MCI and subtle cognitive impairment as well as for Alzheimer’s and neurodegenerative pathologies.

The major strengths of our large-scale study include the community-based study design that engaged rural-dwelling older adults in China who had received no or very limited school education, a sociodemographic group that has been underrepresented in research on dementia and brain aging^46^ and the objective assessment of sleep duration. In addition, using multimodal biomarkers for different brain pathologies assessed with the state-of-the-art techniques (e.g. high-quality 3 T MRI scans and Simoa platform) in MIND-China, we were able to explore the potential mechanisms linking abnormal sleep duration with cognitive phenotypes. However, certain limitations of our study deserve discussion. First, the cross-sectional design of this study did not allow us to establish the temporal associations between sleep duration and outcomes (i.e. cognitive function and biomarkers). Second, sleep duration was assessed using the ECG-based CPC approach that might be affected by heart rate variability regulated via autonomic nervous system during sleep, although the CPC-assessed sleep parameters (e.g. sleep duration) were highly correlated with those assessed using the gold standard polysomnography technique.^25,26^ Third, we were not able to explore the potential pathway of tau pathology in the brain due to the lack of data on plasma phosphorylated-tau181, tau217 or tau231. Furthermore, although plasma and brain Aβ levels were partially correlated, clinical utility of plasma amyloid as a biomarker for the load of amyloid in central nervous system remains to be established partly because peripheral tissues (e.g. liver and kidney) could contribute to circulating amyloid.^47,48^ Finally, cautiousness is needed when generalizing our findings to other populations due to the fact that our study sample was derived from only one rural area in western Shandong Province, China.

In conclusion, our population-based study showed that objectively measured long sleep duration was associated with MCI, naMCI and low function in multiple cognitive domains among dementia-free rural older adults. Furthermore, we revealed that objective long sleep duration was associated with peripheral and neuroimaging biomarkers for Alzheimer’s disease and neurodegeneration (e.g. higher plasma Aβ40 and t-tau, a lower Aβ42/Aβ40 ratio and lower total grey matter volume) but not for cerebral microvascular lesions (e.g. serum adhesion molecules ICAM-1 and VCAM-1 and WMH volume, EPVS and lacunes). This suggests that neurodegeneration may represent common neuropathological pathways underlying both abnormal sleep duration and cognitive impairment in older adults. These findings could bridge the knowledge gap regarding the relationships of abnormal sleep duration with MCI and low cognitive function among rural Chinese older adults and the potential neuropathological mechanisms underlying the associations. Future prospective cohort studies should further investigate the longitudinal relationship of sleep duration with cognitive phenotypes and the potential role of multimodal biomarkers for different brain pathologies in the longitudinal associations.

## Supplementary Material

fcae144_Supplementary_Data

## Data Availability

The data that support the findings of this study are available from the corresponding author, upon reasonable request.
