# Supplementary material for: Association of objective sleep duration with cognition and brain aging biomarkers in older adults
Source: Brain Commun. 2024 Apr 26;6(3):fcae144. doi: 10.1093/braincomms/fcae144 (PMC11098043; doi:10.1093/braincomms/fcae144)
Supplement: fcae144_Supplementary_Data [file fcae144_supplementary_data.pdf]

Supplement Materials

Association of Objective Sleep Duration with Cognition and Brain Aging Biomarkers in Older Adults

Tang S, *et al*.

Supplementary Figure 1. Flowchart of the study participants

Supplementary Figure 2. Multivariable-adjusted spline curves for the associations of CPC-measured sleep duration with mild cognitive impairment (n=2032)

Supplementary Figure 3. Multivariable-adjusted spline curves for the associations of CPC-measured wake after sleep onset with mild cognitive impairment (n=2032)

Supplementary Table 1. Associations between CPC-measured sleep duration and cognitive function among participants with normal cognition (n=1522)

Supplementary Text 1. Methods.

This supplementary material has been provided by the authors to give readers additional information about their work.

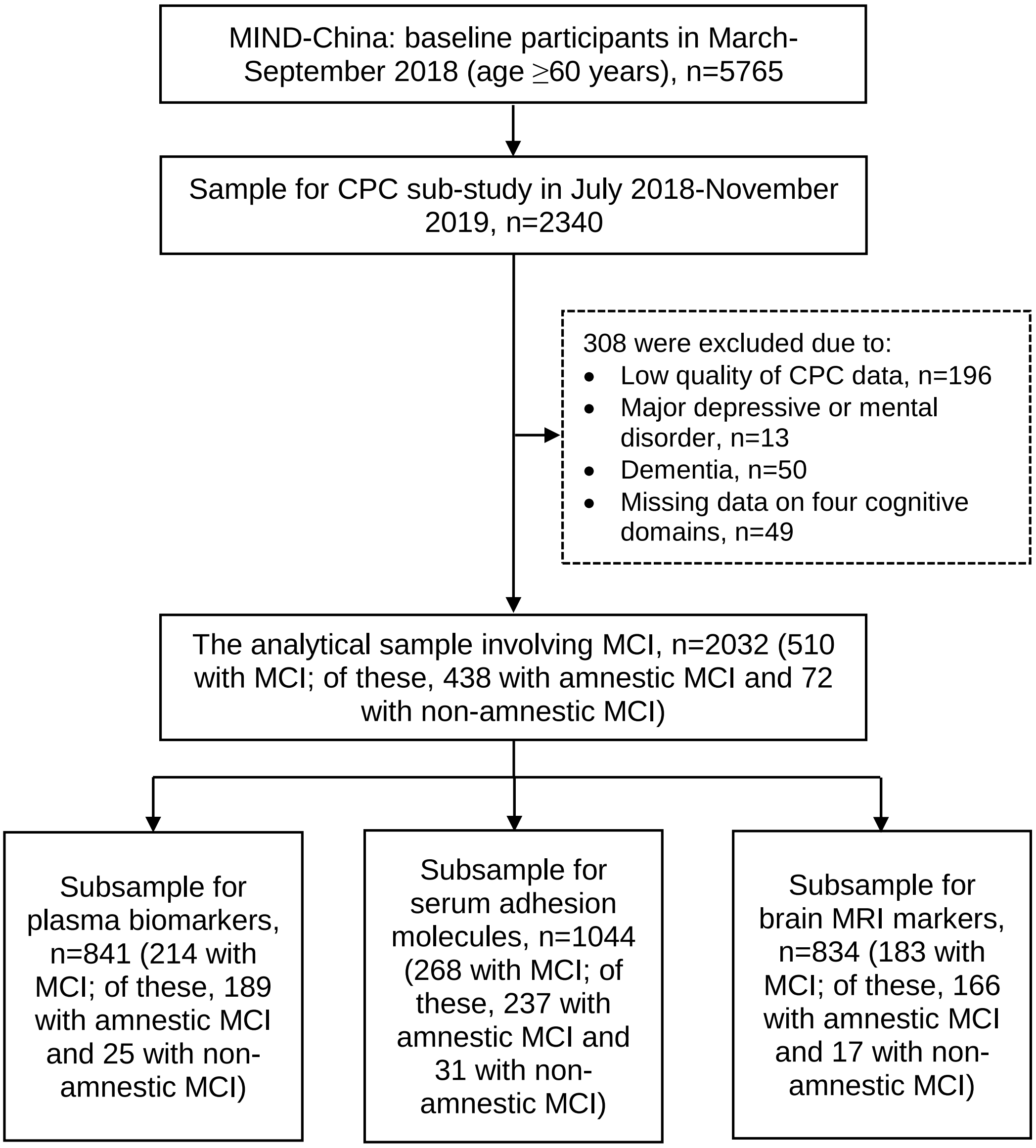

Supplementary Figure 1. Flowchart of the study participants

Abbreviations: CPC, cardiopulmonary coupling; MCI, mild cognitive impairment; MIND-China, the Multimodal Interventions to Delay Dementia and Disability in Rural China; MRI, magnetic resonance imaging; NfL, neurofilament light chain.

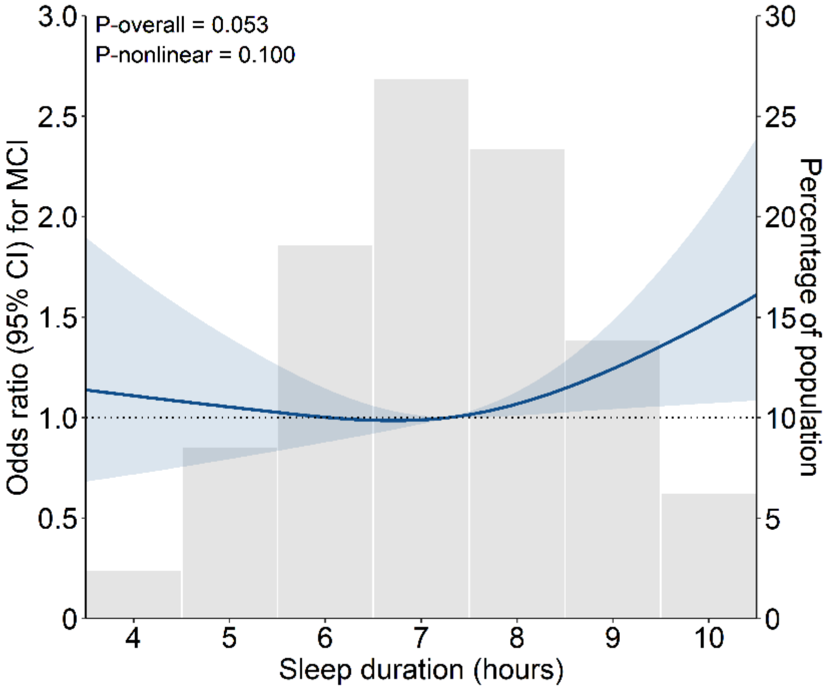

Supplementary Figure 2. Multivariable-adjusted spline curves for the associations of CPC-measured sleep duration with mild cognitive impairment (n=2032)

The nonlinear association of sleep duration with mild cognitive impairment was assessed using restricted cubic spline curve analysis, in which binary logistic regression models were used with three knots at the 10th, 50th, and 90th percentiles of sleep duration. Solid lines represent odds ratio of mild cognitive impairment associated with sleep duration, adjusting for age, sex, education, body mass index, alcohol consumption, smoking, hypertension, diabetes, coronary heart disease, stroke, depressive symptoms, use of hypnotics, and *APOE* genotype. The shaded areas represent the 95% confidence interval. The histogram represents the distribution of study participants.
Abbreviations: CI, confidence interval; CPC, cardiopulmonary coupling; MCI, mild cognitive impairment.

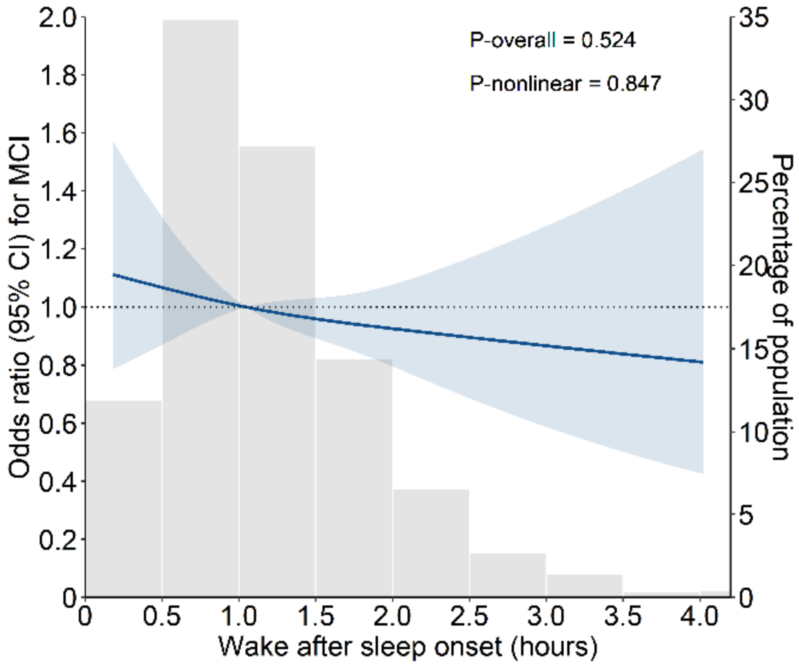

Supplementary Figure 3. Multivariable-adjusted spline curves for the associations of CPC-measured wake after sleep onset with mild cognitive impairment (n=2032)

The nonlinear association of wake after sleep onset with mild cognitive impairment was assessed using restricted cubic spline curve analysis, in which binary logistic regression models were used with three knots at the 10th, 50th, and 90th percentiles of wake after sleep onset. Solid lines represent odds ratio of mild cognitive impairment associated with wake after sleep onset, adjusting for age, sex, education, body mass index, alcohol consumption, smoking, hypertension, diabetes, coronary heart disease, stroke, depressive symptoms, use of hypnotics, and *APOE* genotype. The shaded areas represent the 95% confidence interval. The histogram represents the distribution of study participants. Abbreviations: CI, confidence interval; CPC, cardiopulmonary coupling; MCI, mild cognitive impairment.

Supplementary Table 1. Associations between CPC-measured sleep duration and cognitive function among participants with normal cognition (n=1522)

Sleep duration	No. of participants	β coefficient (95% CI), cognitive z-score			
		Model 1[a]	*p* value	Model 2[a]	*p* value
**Global cognition**[b]					
Sleep duration					
≤6 h	291	-0.04 (-0.10 - 0.02)	.180	-0.04 (-0.11 - 0.02)	.166
6-8 h	772	0.00 (reference)		0.00 (reference)	
>8 h	458	-0.09 (-0.15 - -0.04)	<.001*	-0.09 (-0.14 - -0.04)	<.001*
**Memory**[b]					
Sleep duration					
≤6 h	291	0.06 (-0.03 - 0.15)	.186	0.07 (-0.02 - 0.16)	.147
6-8 h	770	0.00 (reference)		0.00 (reference)	
>8 h	454	-0.03 (-0.10 - 0.05)	.517	-0.02 (-0.10 - 0.05)	.532
**Verbal fluency**[b]					
Sleep duration					
≤6 h	292	-0.10 (-0.19 - -0.002)	.046	-0.10 (-0.19 - -0.003)	.042
6-8 h	770	0.00 (reference)		0.00 (reference)	
>8 h	457	-0.12 (-0.20 - -0.04)	.003*	-0.11 (-0.19 - -0.03)	.005
**Attention**[b]					
Sleep duration					
≤6 h	291	-0.08 (-0.17 - 0.02)	.107	-0.08 (-0.17 - 0.01)	.097
6-8 h	770	0.00 (reference)		0.00 (reference)	
>8 h	458	-0.11 (-0.19 - -0.03)	.006	-0.11 (-0.19 - -0.03)	.009
**Executive function**[b]					
Sleep duration					
≤6 h	291	-0.05 (-0.15 - 0.05)	.325	-0.05 (-0.15 - 0.05)	.291
6-8 h	769	0.00 (reference)		0.00 (reference)	
>8 h	456	-0.11 (-0.19 - -0.02)	.013	-0.10 (-0.19 - -0.02)	.016

[a] Model 1 was adjusted for age, sex, and education. Model 2 was additionally adjusted for body mass index, alcohol consumption, smoking, hypertension, diabetes, coronary heart disease, stroke, depressive symptoms, use of hypnotics, and *APOE* genotype.
[b] Numbers of participants with missing values are 1 for global cognitive z-score, 7 for memory z-score, 3 for verbal fluency z-score, 3 for attention z-score, and 6 for executive function z-score.
*p<.05 after the Bonferroni correction for multiple comparison tests.
Abbreviations: CI, confidence interval; CPC, cardiopulmonary coupling.

Supplementary Text 1. Methods

Data collection and definitions

In MIND-China, the baseline assessment was conducted in March-September 2018, during which trained medical staff collected data via face-to-face interviews, clinical and neurological examinations, neuropsychological testing, and laboratory tests following a structured questionnaire[1]. Briefly, we collected data on demographic factors (e.g., age, sex, and education), lifestyle factors (e.g., alcohol consumption and smoking), health conditions (e.g., hypertension, diabetes, coronary heart disease, and stroke), and use of medications (e.g., antihypertensive, hypoglycaemic, and lipid-lowering agents). Weight and height were measured in light clothes without shoes. Arterial blood pressure was measured on the right arm in a seated position after at least a 5-minute rest using an electronic sphygmomanometer. The 12-lead resting electrocardiogram was recorded by an electrocardiograph and then analysed by a physician. After an overnight fast, peripheral blood samples were taken, and fasting blood glucose and lipids were measured at the clinical laboratory of Yanlou Town Hospital. *APOE* genotyping was performed using the multiple-polymerase chain reaction amplification.

Education was categorized as illiteracy (no formal schooling), primary school, and middle school or above. Body mass index (BMI, kg/m^2) was categorized as ideal (<24.0), overweight (24.0-27.9), and obese (≥28.0), following the criteria recommended for the Chinese adult population. Alcohol consumption and smoking status were categorized as never and ever alcohol drinking or smoking, respectively. Hypertension was defined as arterial blood pressure ≥140/90 mmHg or use of any antihypertensive agents. Diabetes was defined as fasting blood glucose ≥7.0 mmol/L or use of antidiabetic agents or self-reported physician diagnosis of diabetes. Dyslipidaemia was defined as total cholesterol ≥6.22 mmol/L or triglyceride ≥2.27 mmol/L or low-density lipoprotein cholesterol ≥4.14 mmol/L

or high-density lipoprotein cholesterol <1.04 mmol/L or use of hypolipidemic agents. Coronary heart disease was defined according to self-reported history or electrocardiogram examination, including angina pectoris, myocardial infarction, and coronary intervention. Stroke was ascertained according to self-reported history of stroke and neurological examination. Depressive symptoms were assessed using the 15-item Geriatric Depression Scale (GDS-15). The presence of depressive symptoms was defined as a GDS-15 score ≥ 5. *APOE* genotype was dichotomized as carriers vs. non-carriers of the *APOE* ε4 allele[2].

Assessments of sleep characteristics

Data on sleep parameters were collected in July 2018-November 2019 by using the cardiopulmonary coupling (CPC) method. This method uses a continuous single-channel electronic electrocardiogram (ECG) to analyse heart rate variability. Details of the method are available in the Appendix online at www.journalsleep.org. The system enables collecting ECG, actigraphy, body position, and snoring information on multiple nights, and then generates CPC-based sleep parameters (e.g., total sleep time and wake after sleep onset). Activity and body position are assessed by internal accelerometers and gyroscopes.

Neuropsychological assessments and diagnosis of MCI and subtypes

We used a neuropsychological test battery to assess cognitive function, as previously reported[3,4]. Briefly, subjective cognitive decline was assessed via three questions regarding memory problems that the participant experienced in the past year, including difficulty remembering, forgetting what had been planned, and worrying about memory decline [3,5]. The Clinical Dementia Rating Scale (CDR) was used to assess participants' cognitive changes. We used the Mini-Mental State Examination (MMSE) to assess global cognitive function, and the Chinese version of Activities of Daily Living Scale (ADLs) to assess the self-care and instrumental ADLs. We assessed function of the following four cognitive domains[3]: memory was assessed using the Auditory Verbal Learning Test immediate recall, the long-delayed free

recall, and the long-delayed recognition; language was assessed using the Verbal Fluency Test, including animal, fruit, and vegetable categories; attention was assessed with the Trail Making Test-A and Digit Span Forward test; and executive function was assessed using the Trail Making Test-B and Digit Span Backward test. MCI was defined following the Petersen's criteria that were operationalized following an approach similar to that used in the Mayo Clinic Study, as previously described[3,4]: (1) subjective cognitive concern by subjects (responses to the three questions of memory problems or CDR) or informants; (2) objective cognitive impairment evidenced in at least one of the four cognitive domains (from cognitive test battery); (3) essentially preserved functional activities (from the ADLs); and (4) absence of dementia diagnosed according to the DSM-IV criteria. The final judgment about MCI was based on both neuropsychological test scores and a consensus agreement among neurologists specialized in clinical diagnosis, treatment, and care of dementia and cognitive disorders. We further classified MCI into amnestic MCI (aMCI) if the memory domain was impaired, or non-amnestic MCI (naMCI) if there was no impairment in memory function.

MRI data acquisition and processing

Eligible participants were scanned on either the Philips Ingenia 3.0T MR System (Philips Healthcare, Best, The Netherlands) in Southwestern Lu Hospital or the Philips Archiva 3.0T MR System (Philips Healthcare, Best, The Netherlands) in Liaocheng People's Hospital, as described previously[1]. The parameters of core MRI sequences were fully reported elsewhere[1]. We spatially normalized and segmented T1-weighted images and then automatically estimated the total intracranial volume (ICV), volumes of the grey matter, white matter, and hippocampus via the Computational Anatomy Toolbox running on MATLAB (http://dbm.neuro.uni-jena.de/cat12/). The T2-FLAIR images were processed in AccuBrain® (BrainNow Medical Technology Ltd., Shenzhen, Guangdong, China) to acquire the volume of WMHs, as previously described.[6] In brief, AccuBrain® used T2-FLAIR images to calculate

the signal contrast between normal brain tissue and WMHs and set the signal threshold to recognize WMHs. Based on predefined threshold, WMHs were recognized and extracted on T2-FLAIR images. Finally, AccuBrain® refined and localized WMHs using the transformed T1-weighted brain structure mask extracted from our study sample. WMH volume was cubic-root transformed due to right-skewed distribution.[7]

Enlarged perivascular spaces (EPVS) were manually assessed on the axial T2-weighted sequence according to a validated protocol.[8] Briefly, EPVS appear linear when imaged parallel to the course of the vessel, and round or ovoid with a diameter <3 mm when imaged perpendicular to the course of the vessel. The trained rater (M.Z.), who was blinded to the clinical information, visually counted the EPVS in the basal ganglia and centrum semiovale (CSO) bilaterally under the supervision of a senior clinical neurologist (L.S.). The rater first reviewed all MRI slices where the areas of basal ganglia and CSO could be seen, and then counted the number of EPVS on the slice with the highest EPVS number. Global EPVS load was assessed by adding up the EPVS counts in the bilateral basal ganglia and the CSO. The intra-rater correlation coefficient of 0.89 for basal ganglia EPVS and 0.83 for CSO EPVS.

Lacunes were defined as focal fluid-filled cavities of 3-15 mm in diameter; with a hyperintensity ring seen on FLAIR; located in the basal ganglia, subcortical white matter, cerebellum, or brainstem; of irregular or wedge shape; and with irregular margin. Lacunes were dichotomized into ‘absent’ or ‘present’.

References

1. Wang Y, Han X, Zhang X, *et al*. Health status and risk profiles for brain aging of rural-dwelling older adults: Data from the interdisciplinary baseline assessments in MIND-China. *Alzheimers Dement (N Y).* 2022;8(1):e12254.
2. Liang X, Liu C, Liu K, *et al*. Association and interaction of TOMM40 and PVRL2 with plasma amyloid-β and Alzheimer's disease among Chinese older adults: a population-based study. *Neurobiology of Aging.* 2022;113:143-151.
3. Cong L, Ren Y, Wang Y, *et al*. Mild cognitive impairment among rural-dwelling older adults in China: A community-based study. *Alzheimers Dement.* 2023;19(1):56-66.
4. Petersen RC, Roberts RO, Knopman DS, *et al*. Prevalence of mild cognitive impairment is higher in men. *Neurology.* 2010;75:889-897.
5. Jessen F, Amariglio RE, van Boxtel M, *et al*. A conceptual framework for research on subjective cognitive decline in preclinical Alzheimer's disease. *Alzheimers Dement.* 2014;10(6):844-852.
6. Shi L, Wang D, Liu S, *et al*. Automated quantification of white matter lesion in magnetic resonance imaging of patients with acute infarction. *J Neurosci Methods.* 2013;213(1):138-146.
7. Habes M, Erus G, Toledo JB, *et al*. White matter hyperintensities and imaging patterns of brain ageing in the general population. *Brain.* 2016;139(Pt 4):1164-1179.
8. Potter GM, Chappell FM, Morris Z, Wardlaw JM. Cerebral perivascular spaces visible on magnetic resonance imaging: development of a qualitative rating scale and its observer reliability. *Cerebrovasc Dis.* 2015;39(3-4):224-231.
